# Plasma-enabled superhydrophobic coatings on mild steel

**DOI:** 10.1038/s41598-022-26695-w

**Published:** 2023-01-05

**Authors:** Hugo Hartl, Joseph Davies, Geoffrey Will, Kostya Ostrikov, Jennifer MacLeod

**Affiliations:** 1 School of Chemistry and Physics, Centre for Materials Science, Queensland University of Technology (QUT), 2 George Street, Brisbane, QLD 4000 Australia; 2 School of Mechanical, Medical and Process Engineering, Queensland University of Technology (QUT), 2 George Street, Brisbane, QLD 4000 Australia

**Keywords:** Materials science, Chemistry, Materials chemistry, Organic chemistry, Polymer chemistry

## Abstract

This work demonstrates a new pathway to the direct on-surface fabrication of a superhydrophobic surface coating on mild steel. The coating was formed using dielectric barrier discharge (DBD) plasma to convert a liquid small-molecule precursor (1,2,4-tricholorobenzene) to a solid film via plasma-assisted on-surface polymerization. Plasma treatments were performed under a nitrogen atmosphere with a variety of power levels and durations. Samples were analysed by optical and scanning electron microscopy (SEM), energy-dispersive x-ray spectroscopy (EDS), time-of-flight secondary ion mass spectrometry (ToF–SIMS), Raman spectroscopy, optical profilometry, contact angle measurement, and potentiodynamic polarisation tests. Wettability of the films varied with the plasma parameters, and through the inclusion of graphene nanoplatelets in the precursor. High-dose plasma exposures of the nanoplatelet-containing precursor created superhydrophobic films with water contact angles above 150°. Potentiodynamic polarisation tests revealed that the superhydrophobic coating provided little or no corrosion protection.

## Introduction

Superhydrophobic surfaces are low surface energy materials comprising hierarchical motifs of micro- and nano-structures^1–4^. Current fabrication methods for superhydrophobic surfaces include lithography^5–8^, etching^9–12^, self-assembly^13–15^, particle deposition^16^, vapour deposition^16,17^, sol–gel techniques^18–20^, and casting^21^. Of these different approaches, only sol–gel and electrodeposition processes have been used to fabricate superhydrophobic coatings on mild steel^20,22,23^, with many methods focusing on other substrates such as textiles or silicon. Fabricating superhydrophobic surface finishes can be time-consuming and expensive, e.g., due to multi-step processes, long cure times and high temperatures^24,25^.

One important application of superhydrophobic films is in corrosion protection. Corrosion of metals places a large financial burden on many industries, and is therefore of wide interest to mitigate^26.^ There is a strong assumed connection between wettability of the surface and corrosion, with superhydrophobic surfaces usually having notable anti-corrosive properties^27–30^. Superhydrophobic surfaces, which have water contact angles of greater than 150°, limit contact between water and the surface^31^. A survey of the literature reveals a common assumption that as the hydrophobicity of a coating increases, so too will the corrosion resistance that it provides^22,27,32–35^, with superhydrophobic surfaces assumed to offer robust protection against corrosion^26,36–40^. One model for this is based on the hierarchical structure of the surface causing air pockets to form between the corrosive liquid and the substrate, preventing aggressive ions from attacking the substrate by limiting physical interaction^36,37,41–45^. However, this is not always the case, as it has been found that some superhydrophobic films can still provide little to no corrosion protection^46^.

**Figure 1:**
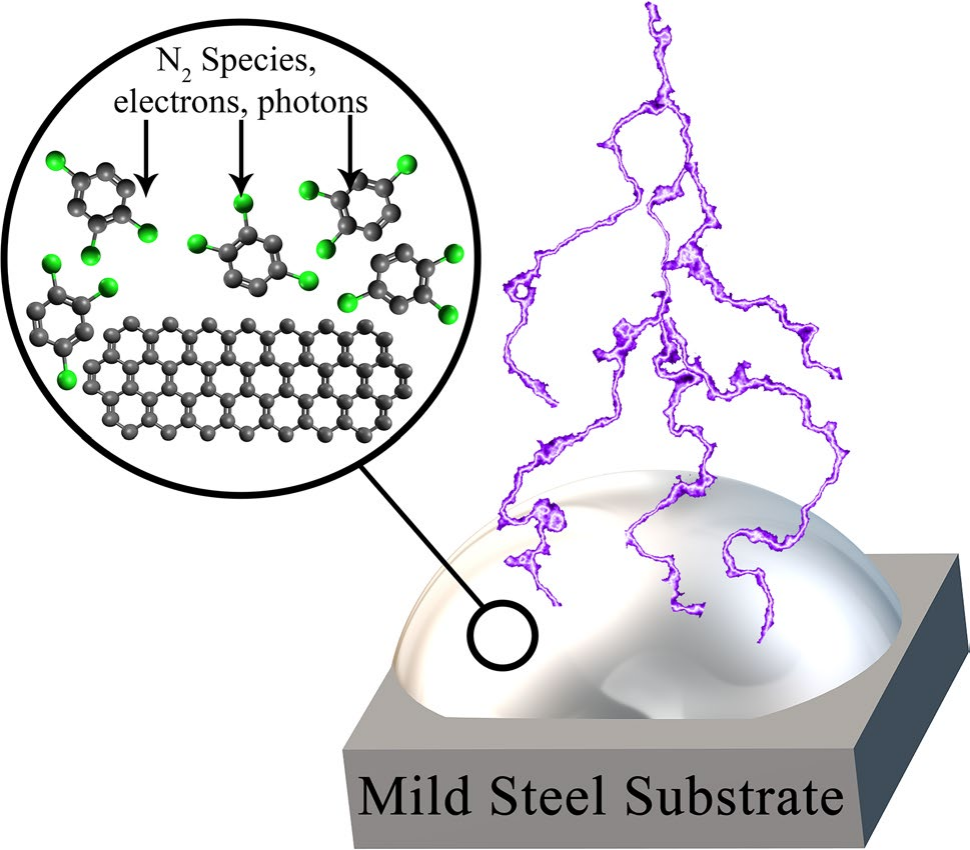
Conceptual illustration of the polymerisation of the TCB + GrNp suspension by plasma exposure.

In this work, a plasma polymerisation method was used for the synthesis of coatings on mild steel. The plasma approach was selected due to the benefits the technique provides such as scalability, energy efficiency, and simple fast processing at room temperature and atmospheric pressure. We have previously shown that plasma treatment promotes a reaction to cleave C–X (carbon–halogen) bonds in 1,2,4-trichlorobenzene (TCB) in the presence of an elemental metal catalyst surface^47^, followed by an oligomerization or polymerisation reaction leading to the formation of a surface-bound solid film. We have shown that the process works on Au and Ni thin films^47^, as well as bulk copper substrates^48^. In this work we demonstrate that the same process can be applied to mild steel (see Fig. 1). Mild steel is an important candidate for corrosion-protective coatings, as it is used in a wide range of applications^49^, despite its intrinsic drawback of readily oxidising (rusting)^50^. We show that the wettability of the films we fabricate on mild steel can be modified through the addition of graphene nanoplatelets (GrNP) in suspension. GrNP are few-layered sheets of sp^2^ carbon^51^, and are naturally hydrophobic^52^. We investigated the effect of both different plasma power levels and dose times for films created with and without GrNP, and characterized the product through optical microscopy and scanning electron microscopy (SEM). Film wettability was assessed through contact angle measurements, and the superhydrophobic film was subjected to corrosion testing. Potentiodynamic polarisation (PDP) tests revealed that the coatings resulted in minor or no corrosion protection, despite their superhydrophobicity. However, despite this failure to provide corrosion protection, we believe that the superhydrophobicity of these coatings could still be useful for applications in areas such as anti-icing^53^.

## Results

### Film morphology

**Figure 2:**
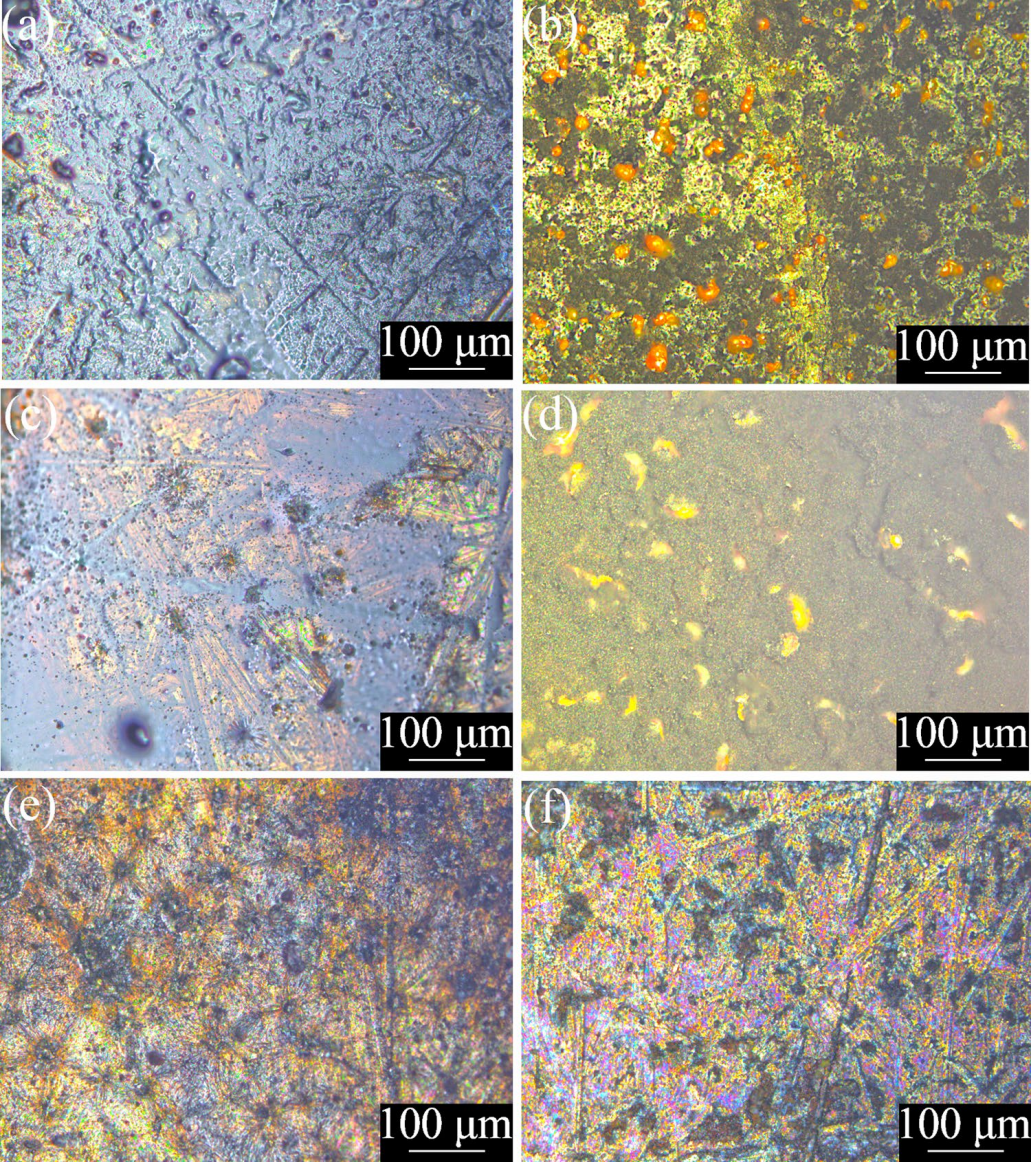
Optical microscopy of the coatings created from a 110 W plasma exposure on a mild steel substrate with an exposure duration of 30 s with (**a**) TCB, (**b**) TCB + GrNp, and an exposure duration of 60 s with (**c**) TCB, (**d**) TCB + GrNp, and an exposure duration of 90 s with (**e**) TCB, and (**f**) TCB + GrNp.

During plasma processing, we observed a liquid-to-solid transition of the TCB, and of the TCB + GrNp suspension, consistent with the formation of higher molecular weight products for all samples, with the resulting films shown in Fig. 2, Figs. A.4 and A.5. For the TCB control samples, the morphologies are similar to those seen previously on other substrates^47^, but occur at lower total energy (power × time) doses than those previously tested. Although we have observed thin film effects in other coatings formed through a similar plasma-enabled process, no such effects were observed here^54^.

As shown in Fig. 2, the TCB + GrNp samples differ in morphology to the TCB coatings, generally appearing rougher, with the 30 s and 60 s exposures showing orange droplets of ~ 20 µm diameter on the surface of the film. We tentatively attribute these to iron oxide formation. These droplets are also seen in the sample produced at 130 W/30 s (see Figs. A.9 and A.10 for optical microscopy of coatings made from other parameters). Optical profilometry confirms that the TCB + GrNp samples have much rougher surfaces, greater maximum feature height, and a significantly increased surface area (seen by the interfacial area ratio, sdr) as compared to the TCB samples (see Table 1).

**Table 1:** RMS roughness (Sq), maximum height (Sz), and developed interfacial area ratio (Sdr) of the coatings produced from a plasma exposure of 110 W 60 s, measured by optical profilometry.

	Sq (μm)	Sz (μm)	Sdr
TCB	46.33	328.39	0.07
TCB + GrNp	439.33	1650.40	28.51

**Figure 3:**
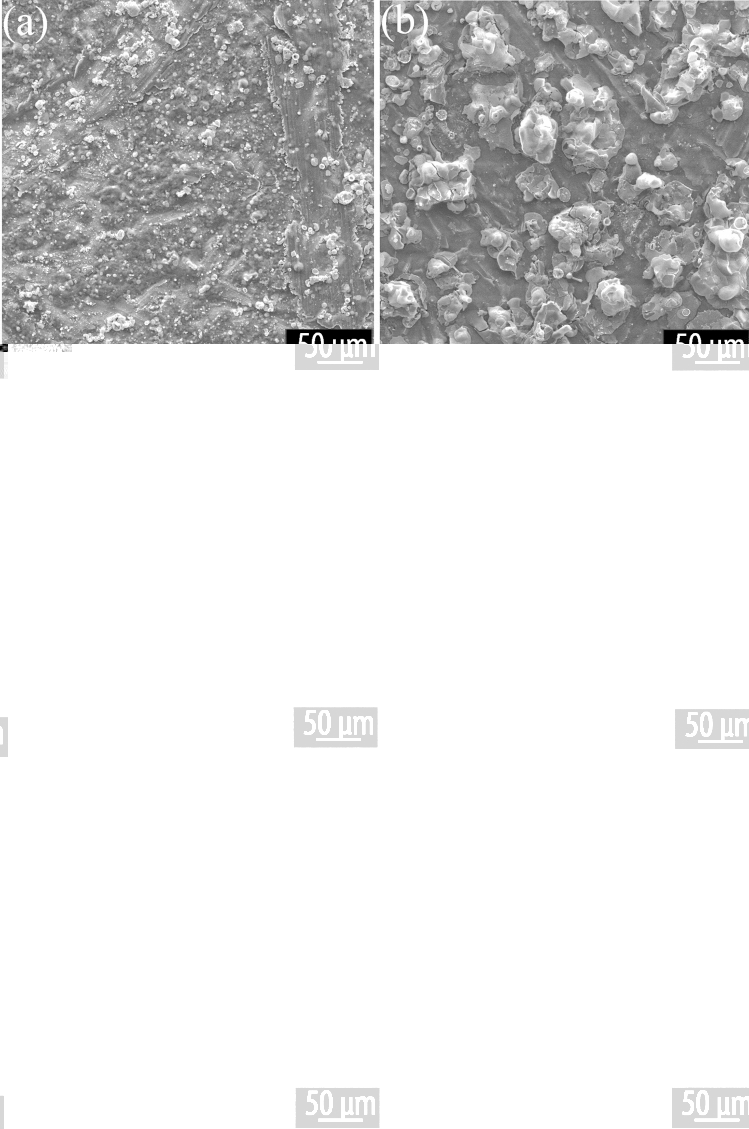
SEM of areas representative of repeatable coatings created from a 110 W plasma exposure on a mild steel substrate with an exposure duration of 30 s with (**a**) TCB, (**b**) TCB + GrNp, and an exposure duration of 60 s with (**c**) TCB, (**d**) TCB + GrNp, and an exposure duration of 90 s with (**e**) TCB, and (**f**) TCB + GrNp.

SEM imaging, shown in Fig. 3, reveals similar features to the optical microscopy, with the TCB + GrNp samples consistently displaying a rougher surface. Whilst the largest features in the TCB coatings are on the order of micrometres, in the TCB + GrNp coatings the feature size is on the order of tens of micrometres. Coatings produced at other plasma power levels also follow this trend (see Figs. A.11 and A.12).

### Water contact angle

**Figure 4:**
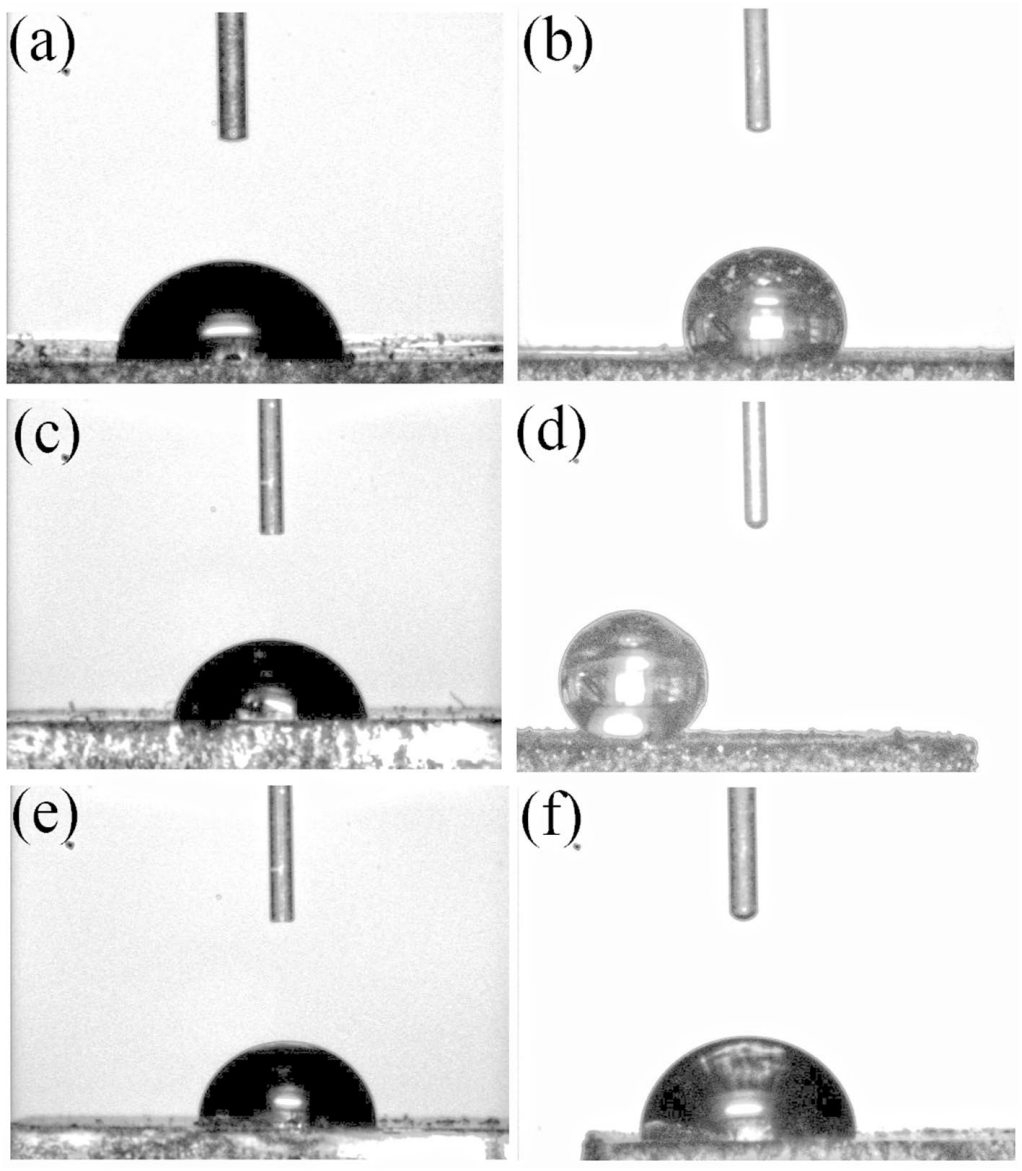
Images from the contact angle setup of water droplets on the coatings created from a 110 W plasma exposure on a mild steel substrate with an exposure duration of 30 s with (**a**) TCB, (**b**) TCB + GrNp, and an exposure duration of 60 s with (**c**) TCB, (**d**) TCB + GrNp, and an exposure duration of 90 s with (**e**) TCB, and (**f**) TCB + GrNp.

The contact angle between a water droplet and a substrate is related to the wettability of the solid, with greater angles indicating lower wettability^55^. Table 2 and Fig. 4 show the contact angle data for all samples created at a plasma power level of 110 W. Water contact angle measurements were taken two days after film creation. The water contact angle was calculated via a method based on the Young–Laplace equation, through ImageJ software^56^. For the TCB samples, the contact angle stays approximately constant with increasing plasma duration, whereas an increase in power correlates with an increase in contact angle (see Table A.4 and Fig. A.13). For the TCB + GrNp samples, the trend is different, with lower power and durations generally more favourable for a high contact angle.

**Table 2:** Contact angles for all TCB and TCB + GrNp coatings produced by a 110 W plasma on a mild steel substrate.

	TCB (°)	TCB + GrNP (°)
30 s	75 ± 5	114 ± 8
60 s	85 ± 5	151 ± 3
90 s	88 ± 3	80 ± 8

The sample with the highest hydrophobicity (110 W/60 s) has such a minimal droplet rolling angle that it is difficult to even measure a static water contact angle as the droplet will readily slide on the most hydrophobic areas of the film surface (see ESI videos). This coating also has a low contact angle hysteresis (CAH) of just 4.3° (see Table A.7), satisfying the requirements to be considered superhydrophobic^57^. The coatings also appear to be quite resistant to acid and alkaline solutions (see Table A.6).

### Film chemistry

**Figure 5:**
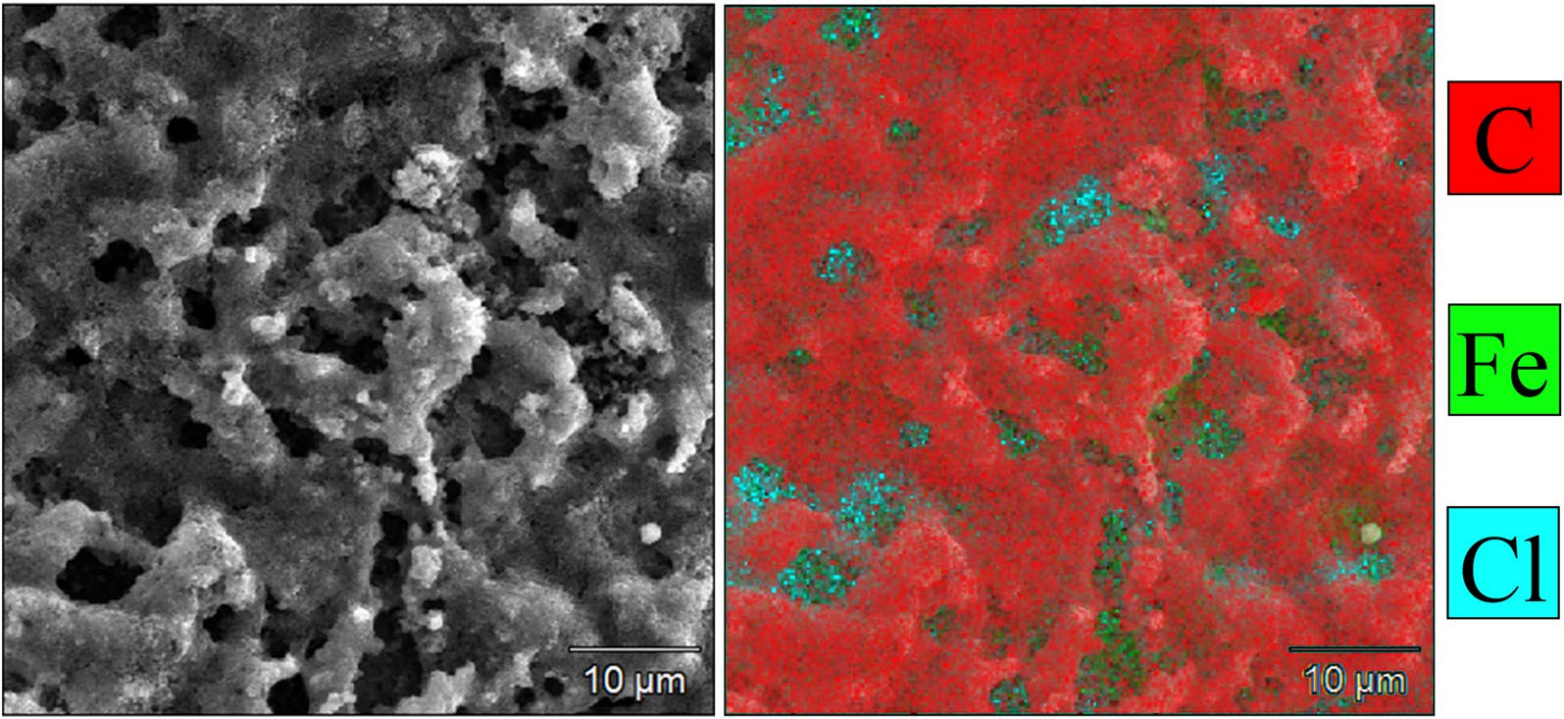
EDS of the TCB + GrNp film produced by the 110 W plasma for 60 s, showing chlorine and iron in the pores of the film.

SEM imaging shows large pores in the surface of the film. Based on energy-dispersive x-ray spectroscopy (EDS) results, the pores are regions where the carbon film is thinner, or the steel substrate is exposed (see Fe signal, Fig. 5). These regions also coincide with the only visible Cl in the film.

ToF–SIMS shows fragments of completely dehalogenated and crosslinked precursor (see Figs. A.7 and A.8). There is no indication of carbon-bonded chlorine in the data. The carbonaceous product should therefore align with the C signal in the EDS in Fig. 5. The only Cl present within the film is bonded to Fe, suggesting that the coinciding Fe and Cl seen in in Fig. 5 are likely a film comprising FeCl_3_ and FeCl_4_, as both fragments were observed in ToF–SIMS, formed under or in the pores of the superhydrophobic carbon surface layer.

X-ray photoelectron spectroscopy (XPS) analysis (see Table A.2) of the films shows incorporation of nitrogen from the plasma atmosphere, and a generally lower Cl:C ratio than the intact molecule. This suggests that chlorine has been cleaved from the TCB. Fourier-transform infrared spectroscopy (FTIR) analysis (see Fig. A.14) shows C–O and C=O bonding, suggesting that atmospheric oxygen has been incorporated into the film.

### Corrosion

The sample that produced the highest water contact angle (110 W/60 s) was selected for corrosion testing, under the assumption that it should have the highest level of corrosion protection^22,32–35^. A neat TCB coating (without GrNp), fabricated through the same 110 W/60 s plasma, was also tested. Finally, a bare steel substrate, exposed to the same plasma parameters as the two coated samples, acted as a control sample.

**Figure 6:**
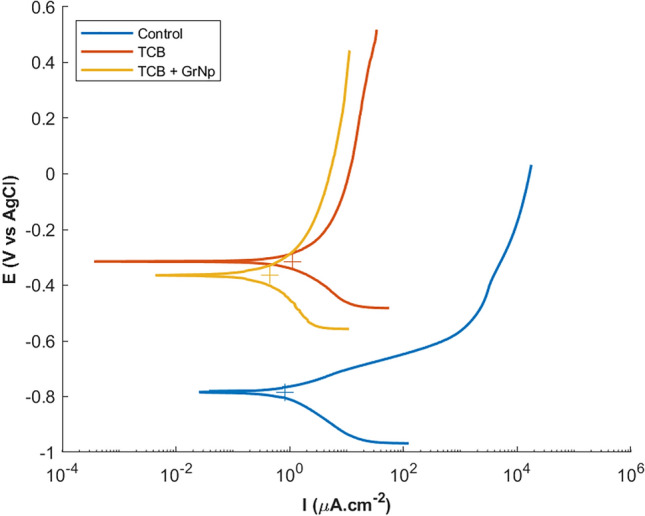
Tafel diagrams of PDP with the locus of (I_corr_, E_corr_) shown as crosses.

Corrosion behaviour in NaCl solution (3.5% in water) was investigated with potentiodynamic polarisation (PDP). Polarisation curves are shown in Fig. 6 and summarised in Table 3. Current densities of TCB and TCB + GrNp during the anodic polarisation were much steeper than would be expected from iron oxidation under activation control, indicating significant influence from a passivating layer^46^. Under these conditions, anodic current densities are the sum of the corrosion reaction and passivating mechanisms, which cannot be deconvoluted. Cathodic current densities correspond to the reduction of oxygen and hydrogen as part of the corrosion reaction^58^. A cathodic polarisation curve was fit to the linear region and the corrosion current (I_corr_) was obtained from the intersection with the corrosion potential E_corr_. The locus of (I_corr_, E_corr_) is shown as a cross in Fig. 6. The results revealed that the TCB and TCB + GrNp films provided little or no corrosion protection, despite them being superhydrophobic. Similar hydrophobic coatings described in the literature including their water contact angle and corrosion protection performance have been tabulated in the ESI (see Table A.5).

**Table 3:** Summary of corrosion current, corrosion potential and cathodic Tafel slopes estimated from two replicates.

Treatment	E_corr_ (V vs AgCl)	I_corr_ (μA / cm^2^)	B_c_ (mV / decade)
Control	−0.8 ± 0.1	0.9 ± 0.1	151 ± 11
TCB	−0.3 ± 0.1	1.4 ± 0.3	185 ± 27
TCB + GrNp	−0.4 ± 0.1	0.5 ± 0.1	206 ± 39

## Discussion

The deposited films vary considerably in morphology according to the plasma exposure, and with the presence/absence of GrNp. The samples containing GrNp have a surface that is approximately 10 times as rough, and five times as thick as the samples without. The surface area of the films containing GrNp is significantly higher than those without, at almost 30 times greater. The superhydrophobic sample also appears roughest under SEM imaging (see Fig. 3), and has the clearest Raman signature for graphene (see Fig. A.6).

For the TCB samples, the wettability of the films appears to decrease with increasing plasma duration or power. This is similar to what we have previously observed^47^, likely due to increasing roughness of the surface with increasing plasma dose. For the TCB + GrNp samples, however, the trend is very different. A dose (110 W/60 s) in the middle of the sample space we investigated appears to be optimal to create a superhydrophobic surface. We show here that it was possible to create a film with a very high contact angle with only a minute of plasma exposure. This same film also has a very low (close to zero) sliding angle (see ESI video), and a contact angle hysteresis of 4.3°.

Coating the steel with TCB + GrNp and TCB films results in a decreased corrosion current compared to the control sample. We assume the corrosion current is coming from regions of steel exposed through the porous films, which represents a reduced surface area relative to the control sample due to the presence of the coating. As shown in Fig. 5, EDS imaging indicates that pores extend through the full thickness of the TCB + GrNp film, revealing a FeCl_x_ film covering the steel substrate. We attribute the creation of the pores in the film to the liquid–solid transition that the precursor undergoes during plasma exposure. During the plasma exposure, molecules of the precursor contact the substrate, and a surface-catalysed dehalogenation reaction occurs, cleaving halogens and bonding products by C–C coupling. We hypothesise that these bonded fragments then navigate the precursor droplet by convection, to be deposited at the top of the coating through ‘volcano’-like systems. As all precursor solidifies, these ‘volcano’-like systems are left hollow, forming pores.

A droplet on a rough surface can be in one of two wetting states, the Cassie–Baxter state or the Wenzel state^13^. In the Wenzel state, the droplet fully contacts the substrate. The Cassie–Baxter state occurs when the contact of a droplet with a surface is limited by trapped air pockets. These air bubbles that are formed under the droplets of water on a surface are a known phenomenon in superhydrophobic coatings, termed plastrons^59^. When these surfaces are submerged (such as during corrosion testing), the loss of plastrons is known to occur under certain conditions relating to the water pressure and the structures at the surface of the coating^60^. However, as the surface features in our coatings are non-identical, these conditions could vary over the surface. This means that plastron breakdown could occur locally rather than surface-wide, which could lead to localized water-substrate contact. The FeCl_3_ observed within the pores of the film by EDS and ToF–SIMS is hydrophilic. The water contact on the surface could therefore also exist in a hybrid state, the Wenzel-Cassie state, wherein the water partially contacts the surface, and is partially supported by air pockets. Therefore, the Wenzel or Wenzel–Cassie state could lead to corrosion of the substrate directly through pores in the coating, offering a plausible mechanism for the poor corrosion protection that these coatings provide.

Despite the lackluster corrosion protection results measured, these films still present an opportunity for use in other applications. For example, these coatings could be used in anti-icing applications. The coatings were also shown to be resistant to acid and alkali solutions, suggesting possible uses in harsh environments. We note that this process has potential for upscaling, for example in a large roll-to-roll system^61^. The fabrication process comprises only a single step and occurs at room temperature and ambient pressure, making it an excellent candidate to scale-up. The process is also energy efficient, producing a superhydrophobic coating on the 10 × 10 mm^2^ substrate in just 60 s at 110 W. Further cost analysis for scale-up can be seen in the ESI.

## Conclusion

We have demonstrated the use of room-temperature, atmospheric-pressure plasma to create a superhydrophobic film on the surface of mild steel. We specifically investigated the incorporation of GrNp into a liquid-phase precursor, 1,2,4-trichlorobenzene, creating a solid, thin-film product.

Different plasma parameters and the incorporation of GrNp led to different film morphologies. The wettability of the films is dependent on the plasma parameters and incorporation of GrNp, with hydrophobicity reaching an observed maximum for the GrNP-containing sample fabricated with a plasma dose of 110 W for 60 s. Potentiodynamic polarisation testing revealed that neither the TCB nor the superhydrophobic TCB + GrNp films offered significant corrosion protection. The films may be more suitable for applications in areas such as anti-icing.

## Methods

As in our previous research^4,47,48,54^, plasma treatments were performed using a dielectric barrier discharge (DBD) apparatus at room temperature (see Fig. A.1). Samples were placed in a quartz chamber, equipped with a high-voltage AC generator (CTP-2000K, Corona Laboratory). Nitrogen was flowed through the chamber as the working gas to minimize the oxidation of the surface, at a flow rate of 200 mL/min (measured by a Restek ProFLOW 6000). The DBD chamber has an internal volume of 20 mL, therefore this equates to a total atmosphere replacement time of six seconds. The chamber is at atmospheric pressure, as the outlet of the DBD chamber is open to atmosphere. The distance between the top of the DBD chamber and the steel substrate was ~ 6 mm.

For synthesis experiments, mild steel substrates were polished by 500 grade sandpaper until all surface oxidation was removed, followed by 1200 grade sandpaper for one minute in a figure-eight movement, immediately prior to deposition of coating. 1,2,4-trichlorobenzene (TCB, 99% Sigma Aldrich) can be dehalogenated to form a hydrophobic solid film following plasma exposure at a reactive metal surface^47^. In the present work, we investigate the effect of adding graphene nanoplatelets (GrNp, 99 wt%, < 4 layers, 1–2 μm lateral dimensions, Cheap Tubes, USA) to the TCB. 8 μL of TCB or 8 μL of the TCB + GrNp suspension was then deposited on each stationary 10 mm × 10 mm mild steel substrate (see Table A.3 for X-ray fluorescence (XRF) data of the mild steel, showing atomic concentrations) using an auto-pipette. The precursor wetted each substrate fully, creating a convex droplet (see Fig. A.3).

Following the process from our previous research^4,47,48,54^, each sample was treated with plasma immediately after precursor deposition. For each sample, we used the same peak–peak generator output voltage of ~ 30 kV, as measured by a Rigol DS6104 digital oscilloscope (see Fig. A.2 for a typical waveform). The output power and current of the plasma should increase linearly with increasing input power, as the output voltage and frequency were kept constant at ∼30 kV and ∼40 kHz. The operating frequency was adjusted for the optimum DBD generation for the dielectric barrier, which is characterised by the dielectric constant and thickness. High voltage was achieved using a 0–250 V input voltage regulator feeding the CTP-2000K. The plasma characteristics, including waveform and power output, are consistent with those reported by others using the same equipment^62^. All of the plasma conditions investigated in this work are reported in Table A.1 in the ESI. We tested both differing durations of plasma exposure times and varying input powers. Following testing of different concentrations varying from 1 to 10 wt%, and plasma power levels varying from 90 to 130 W, a concentration of 1 wt% at 110 W was selected for detailed investigation, as this provided the highest water contact angle and most homogenous films.

The morphology of the surface product was investigated by optical microscopy (Leica DM6000M), and scanning electron microscopy (SEM, TESCAN MIRA3). The contact angle of a water droplet with the surface of the films was measured by a drop shape analyser (FTÅ200) using a rate of 2 μL s^−1^. Profilometry was performed using a Zeta 300 optical profiler, over both a 3140 µm × 2471 µm region, and a 157 µm × 124 µm region. Energy-dispersive x-ray spectroscopy (EDS, TESCAN MIRA3) imaging was used to investigate the film chemistry, and was performed at a 10 kV acceleration voltage, with a scan time of 500 s. Following the same process of our previous analysis of similar films^4,48,54^, time-of-flight secondary ion mass spectrometry (ToF–SIMS) was used to investigate the film chemistry. ToF–SIMS data were acquired using an IONTOF M6 instrument (ToF–SIMS, IONTOF GmbH) equipped with a reflectron time-of-flight analyser and Bi/Mn primary-ion source. Bi_3_^+^ cluster ions were selected from the pulsed primary-ion beam for the analysis and bunched to minimise the pulse length, thereby maximising the mass resolution (Δm/m = 7000 for C_2_H_3_^+^ and C_2_^-^). The primary-ion dose was limited to 10^11^/cm^2^, which is below the static limit. Data were acquired from 500 µm × 500 µm regions of the sample whilst flooding with low-energy electrons (21 eV) to compensate for surface charging. A cycle time of 140 µs provided an accessible mass range of m/z 0–1000 for the secondary ions. Data were acquired in both positive and negative polarity, and the mass scales calibrated using peaks attributed to hydrocarbon ions (C^+^, CH^+^, CH_2_^+^, C_2_H_3_^+^, C_3_H_5_^+^, C_(4–7)_H_7_^+^; C^−^, CH^−^, CH_2_^−^, C_(2–4, 6, 7)_^−^). The pressure in the analysis chamber during data acquisition was at, or below, 3 × 10^–9^ mbar.

Corrosion behaviour of the TCB and TCB + GrNp coatings produced at 110 W/60 s were investigated in a NaCl solution (3.5 wt%, in water) with a three-electrode system shielded by a Faraday cage using a BioLogic SP200 potentiostat. Platinum mesh and saturated Ag/AgCl were used as counter and reference electrodes. The working electrode had an area of 0.13 cm^2^ of sample surface exposed to the test solution, sealed by an o-ring. Mild steel control samples were polished by 1200 grade sandpaper immediately prior to testing. Electrochemical methods were performed after two hours of immersion so that open circuit potential (OCP) could stabilise before the test. All tests were conducted in duplicate (n = 2), with each replicate test occurring on a different day.

## Supplementary Information

Supplementary Information.

Supplementary Video 1.

Supplementary Video 2.

## Data Availability

Supplementary information accompanies this paper at http://www.nature.com/scientificreports.
