# Supplementary material for: Plasma-enabled superhydrophobic coatings on mild steel
Source: Sci Rep. 2023 Jan 5;13:255. doi: 10.1038/s41598-022-26695-w (PMC9816161; doi:10.1038/s41598-022-26695-w)
Supplement: Supplementary file 1 — Supplementary Information. [file 41598_2022_26695_MOESM1_ESM.pdf]

Electronic Supplementary Information

for

Plasma-Enabled Superhydrophobic Coatings on Mild Steel

Hugo Hartl[1], Joseph Davies[2], Geoffrey Will[2], Kostya (Ken) Ostrikov[1], and Jennifer MacLeod[1,*]

[1] School of Chemistry and Physics and Centre for Materials Science, Queensland University of Technology (QUT), 2 George Street, Brisbane, QLD, Australia 4000

[2] School of Mechanical, Medical and Process Engineering, Queensland University of Technology (QUT), 2 George Street, Brisbane, QLD, Australia 4000

[*]jennifer.macleod@qut.edu.au

Contents

Plasma Parameters 2

Photograph of Plasma Setup 2

Typical Oscilloscope Trace for the 90 W Plasma 3

Photograph of Precursor Droplet on Mild Steel Sample 3

Photographs of all Samples Made 4

XPS of TCB Samples 5

XRF of Mild Steel Substrate 6

Raman Spectroscopy 6

ToF-SIMS 8

Optical Microscopy of Films Produced from Other Plasma Parameters 10

SEM of Films Produced from Other Plasma Parameters 12

Water Contact Angle of Films Produced from Other Plasma Parameters 14

FTIR of TCB+GrNp Film 15

Literature of Superhydrophobic Films 16

Water Contact Angle of Films after Exposure to Acid/Alkaline Solutions 16

Further Water Contact Angle 16

Cost Analysis of Scale-up 17

Plasma Parameters

Table A.1 Plasma conditions investigated in this research.

	TCB	TCB+GrNp
90 W	30, 60, 90 s	30, 60, 90 s
110 W	30, 60, 90 s	30, 60, 90 s
130 W	30, 60, 90 s	30, 60, 90 s

Photograph of Plasma Setup

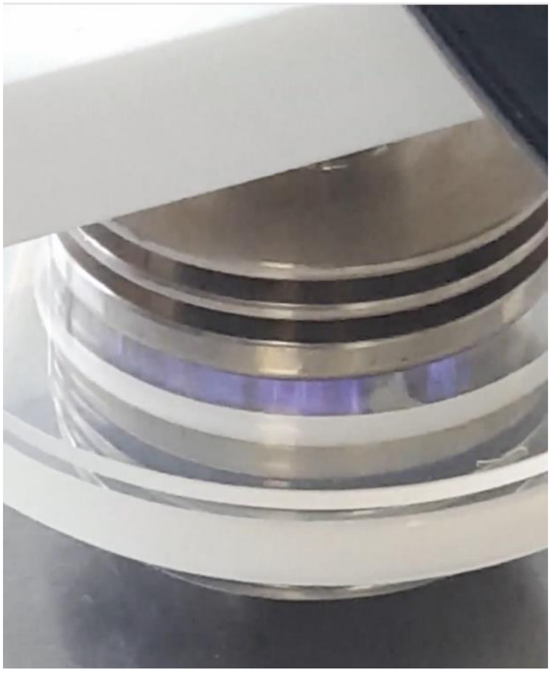

Figure A.1 Photograph of the dielectric barrier discharge plasma apparatus used in this work.

Typical Oscilloscope Trace for the 90 W Plasma

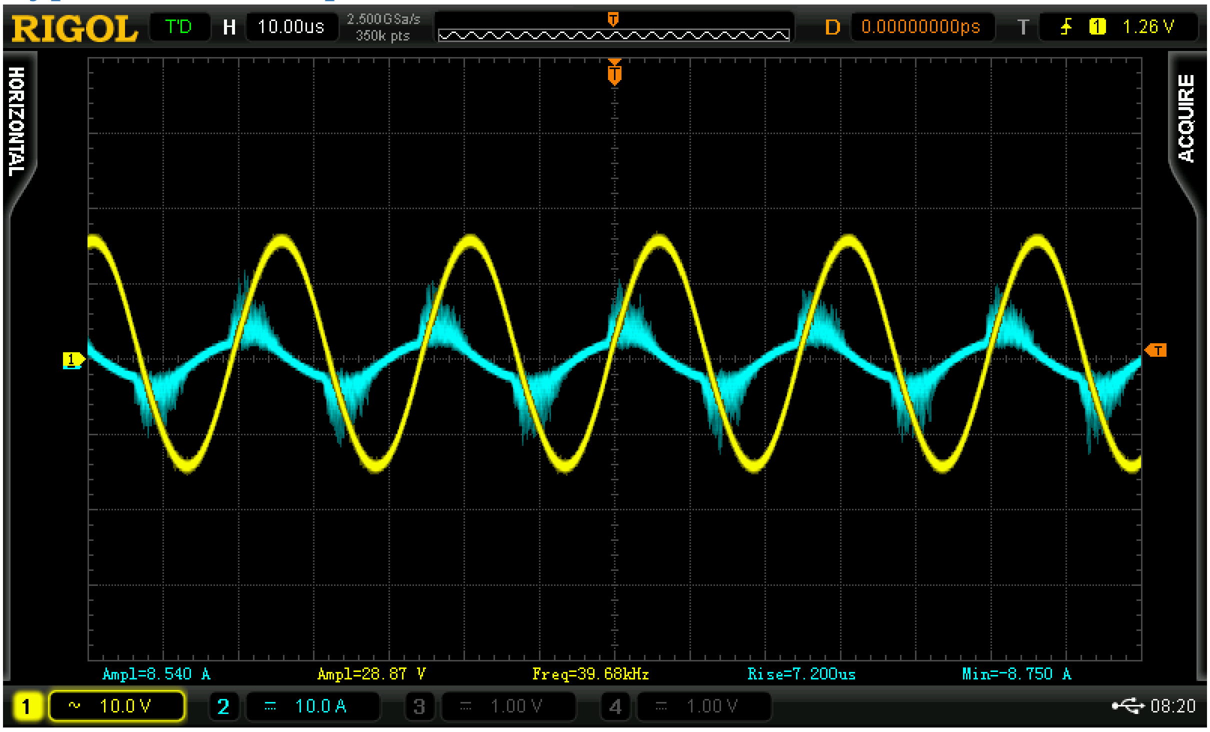

Figure A.2 Screen capture of the typical waveform of a 30 kV plasma produced in this experimental setup from a 90 W input power. The yellow trace displays voltage, and the blue trace shows current.

Photograph of Precursor Droplet on Mild Steel Sample

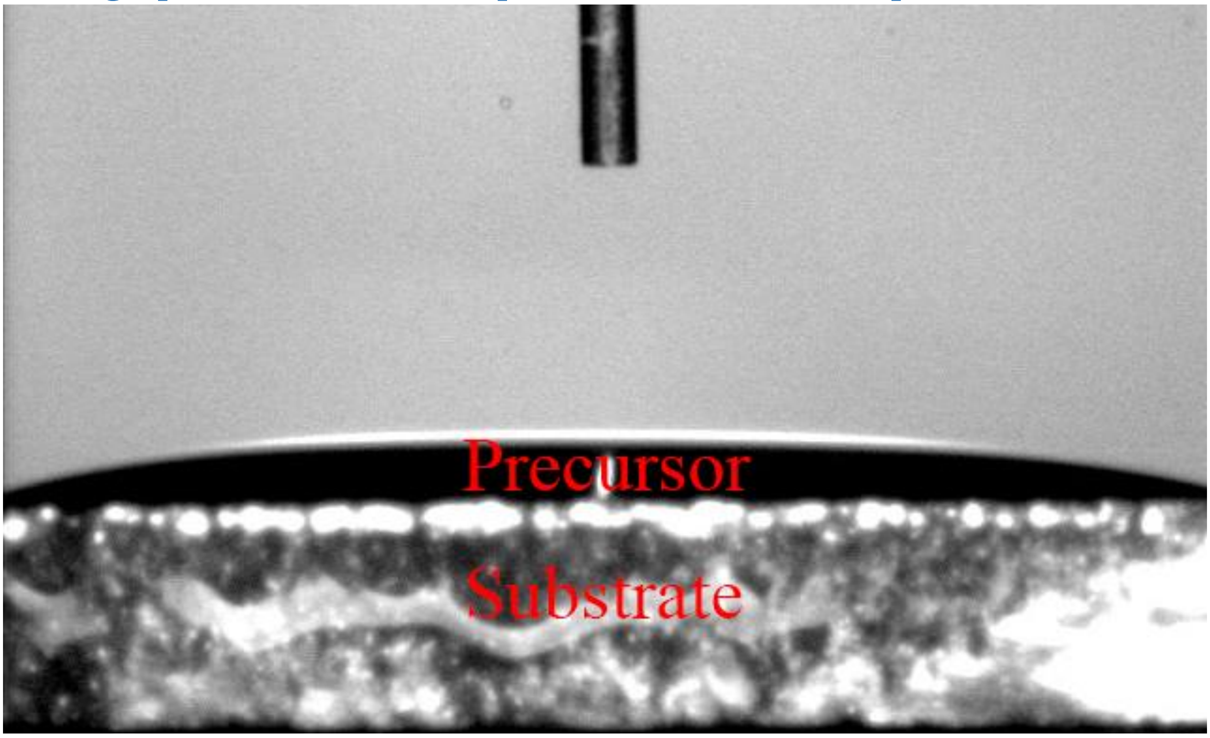

Figure A.3 Photograph of a TCB liquid droplet on mild steel substrate following deposition, showing that the droplet wets the surface of the metal.

Photographs of all Samples Made

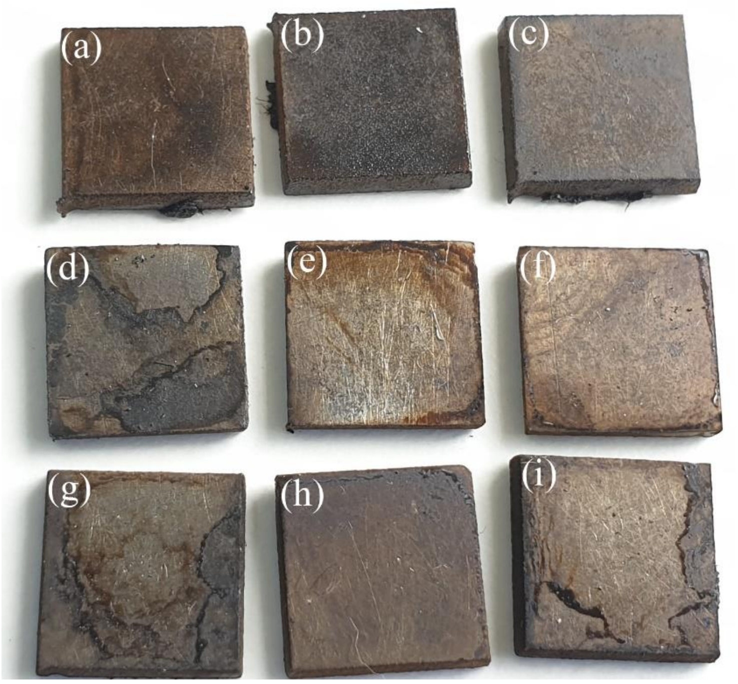

Figure A.4 All of the TCB coatings created with exposures of (a) 90 W 30 s, (b) 90 W 60 s, (c) 90 W 90 s, (d) 110 W 30 s, (e) 110 W 60 s, (f) 110 W 90 s, (g) 130 W 30 s, (h) 130 W 60 s, (i) 130 W 90 s.

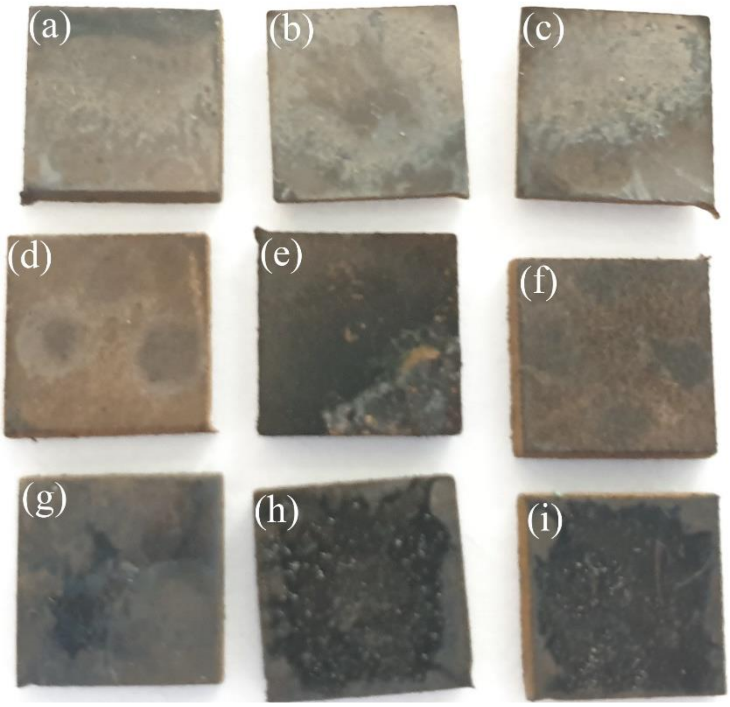

Figure A.5 All of the TCB+GrNp coatings created with exposures of (a) 90 W 30 s, (b) 90 W 60 s, (c) 90 W 90 s, (d) 110 W 30 s, (e) 110 W 60 s, (f) 110 W 90 s, (g) 130 W 30 s, (h) 130 W 60 s, (i) 130 W 90 s.

XPS of TCB Samples

X-ray photoelectron spectroscopy (XPS, Kratos Axis Supra) using an Al Kα source (1486.87 eV) and with 225 W emission power was used to characterize the films. A step size of 1.0 eV for 120 s was used for wide scans. All measurements were performed at room temperature. XPS analysis was performed in CasaXPS Version 2.3.19 [1].

Table A.2 XPS-measured elemental composition (atomic percentage) of TCB films irradiated by a plasma under nitrogen atmosphere at different power levels and durations.

Sample	O (%)	C (%)	N (%)	Fe (%)	Cl (%)	Cl : C
Unreacted TCB (stoichiometric)	-	66.6	-	-	33.3	0.50
90 W 30 S	10	63	6	1	20	0.32
90 W 60 S	20	57	4	5	14	0.25
90 W 90 S	24	54	3	7	13	0.24
110 W 30 S	3	65	3	0	29	0.45
110 W 60 S	3	58	9	0	30	0.52
110 W 90 S	9	53	21	1	17	0.32
130 W 30 S	2	62	7	0	28	0.45
130 W 60 S	4	55	12	0	29	0.53
130 W 90 S	5	60	10	1	25	0.42

XRF of Mild Steel Substrate

X-ray fluorescence (XRF, SPECTRO XEPOS) was used to chemically characterize the mild steel substrates.

Table A.3 XRF of the mild steel used as substrates for the films, showing atomic concentrations.

	Concentration (wt%)	± (wt%)
Si	0.017	0.001
P	0.014	0.001
Ti	0.002	0.001
Cr	0.046	0.001
Mn	0.389	0.001
Fe	99.471	0.040
Ni	0.037	0.001
Cu	0.008	0.001
Zn	0.002	0.001
As	0.002	0.001
Mo	0.008	0.001
Ta	0.005	0.001
Total	100.00	

Raman Spectroscopy

Raman spectroscopy with an excitation wavelength of 532 nm was used to investigate the structure of the GrNp in the films (Renishaw inVia). Raman spectroscopy was performed to compare the raw GrNp powder signature to that of all created TCB+GrNp films, displayed in Fig. A.5. For this, a range of 0-3000 cm^{-1} was used, to display the three characteristic peaks of graphene.

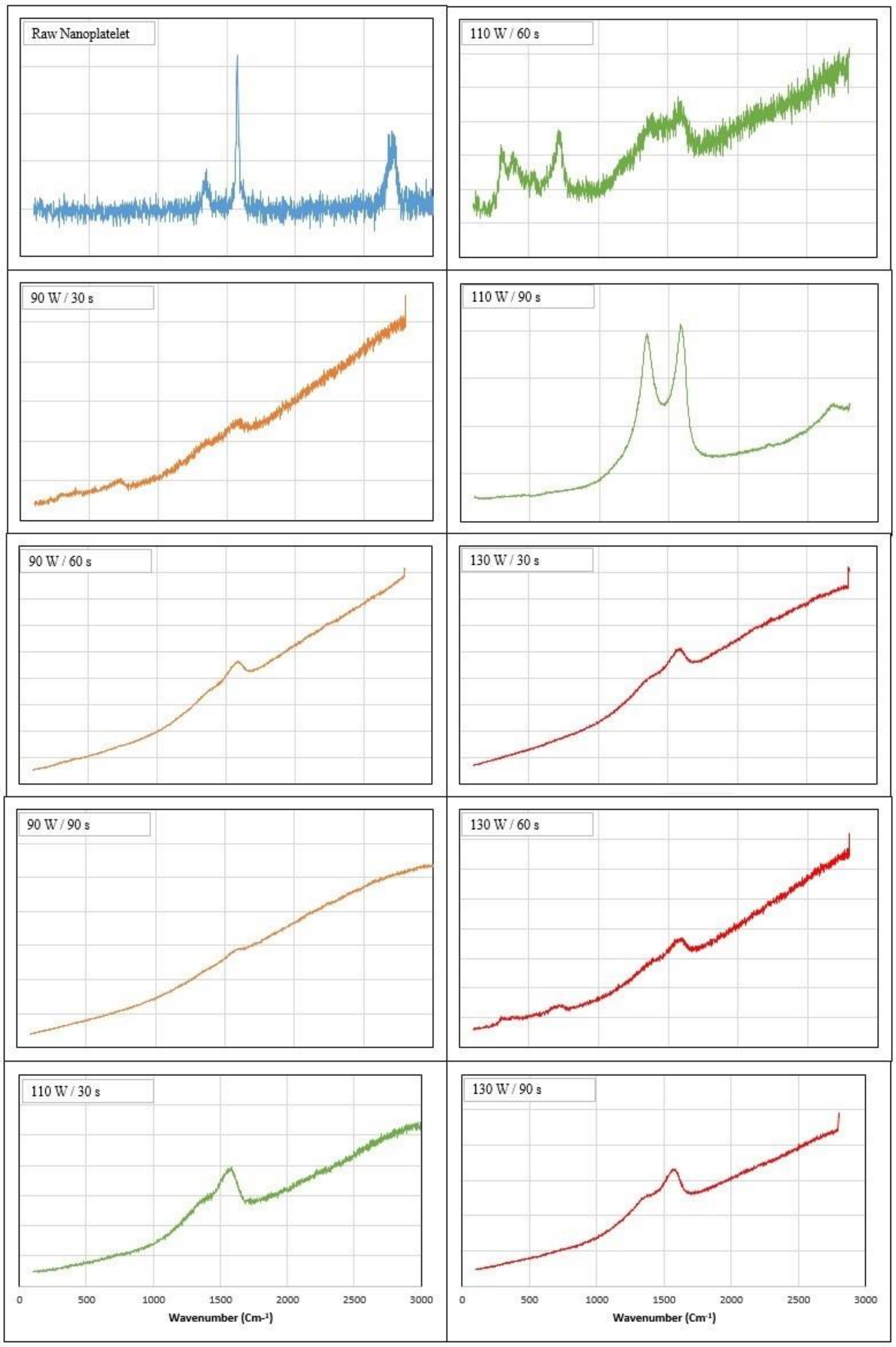

Figure A.6 Raman spectroscopy of all the samples, compared to the raw GrNp powder.

ToF-SIMS

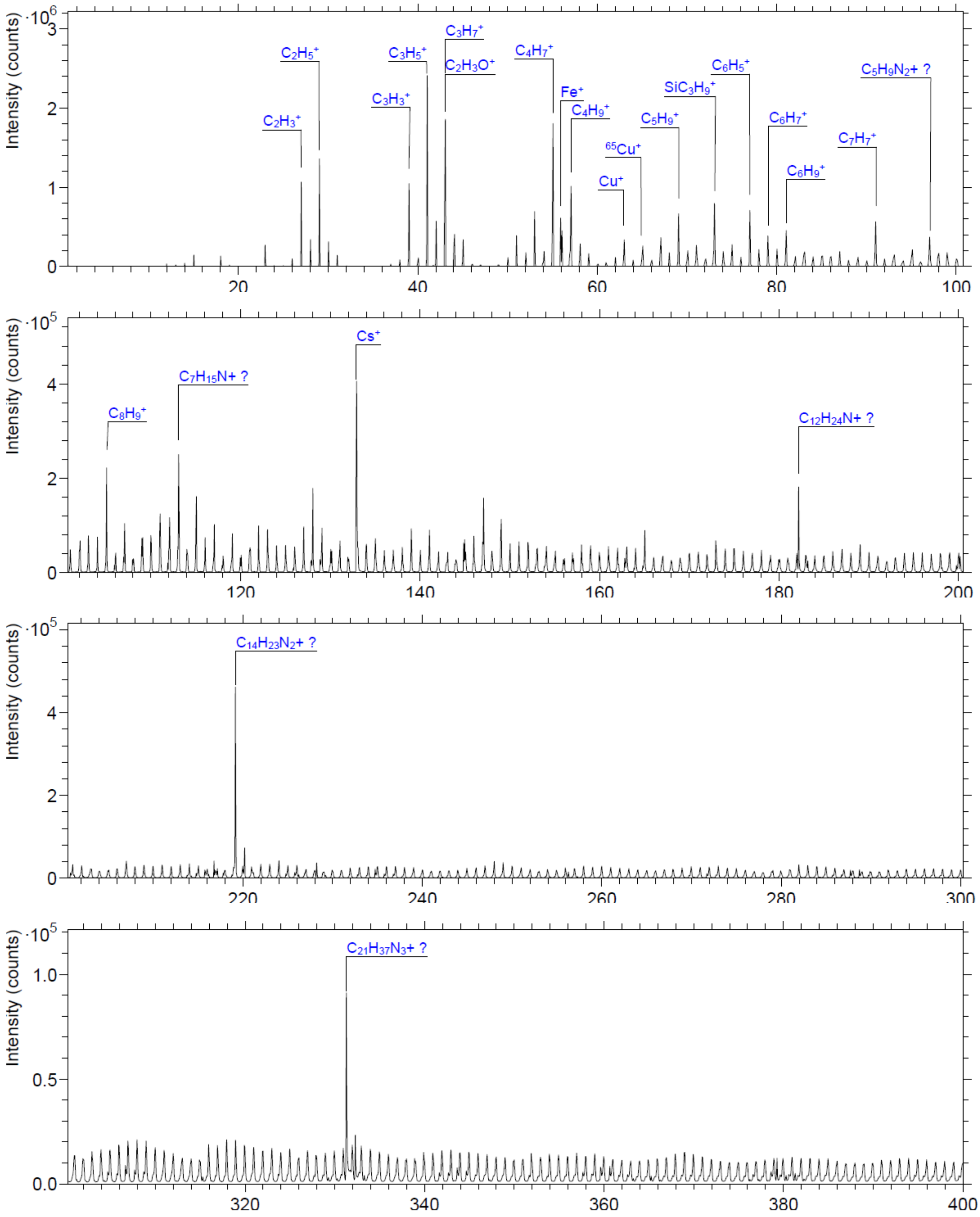

Figure A.7 Positive polarity ToF-SIMS data of the TCB film produced at 110 W plasma for 60 s.

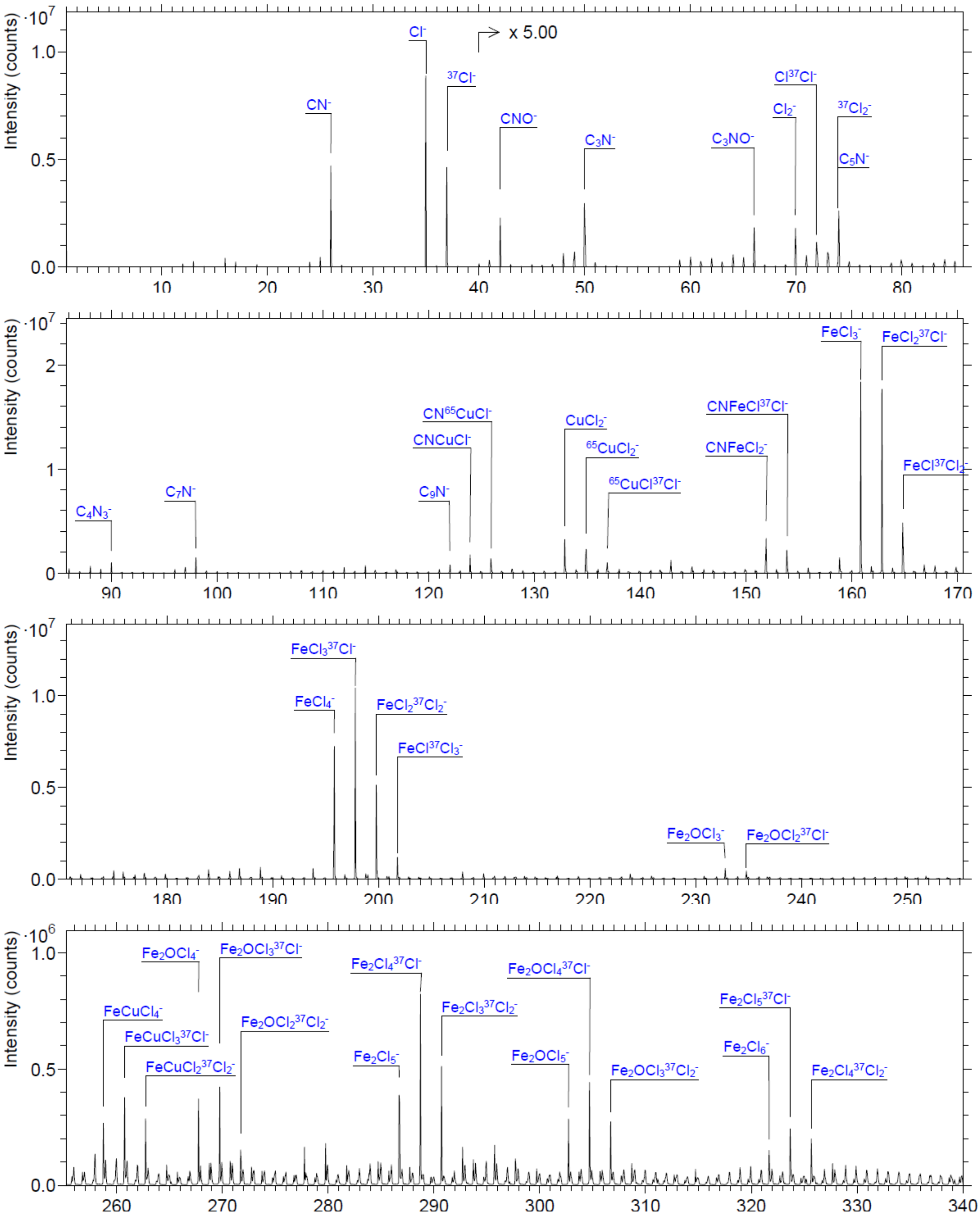

Figure A.8 Negative polarity ToF-SIMS data of the TCB film produced at 110 W plasma for 60 s.

Optical Microscopy of Films Produced from Other Plasma Parameters

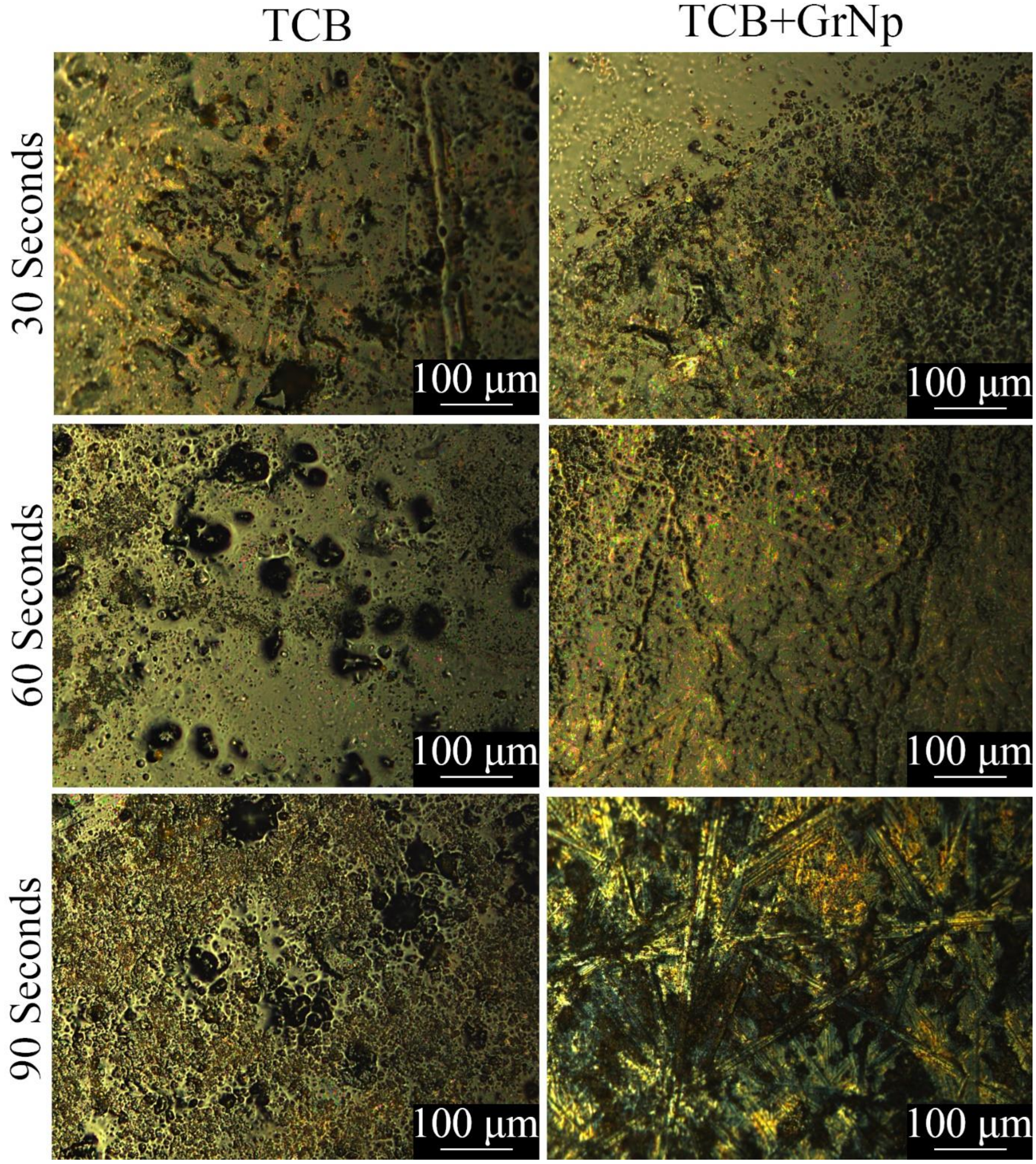

Figure A.9 Optical microscopy of plasma reacted TCB and TCB+GrNp on mild steel substrate, at 90 W for varying durations (specified along the vertical axis of the figure).

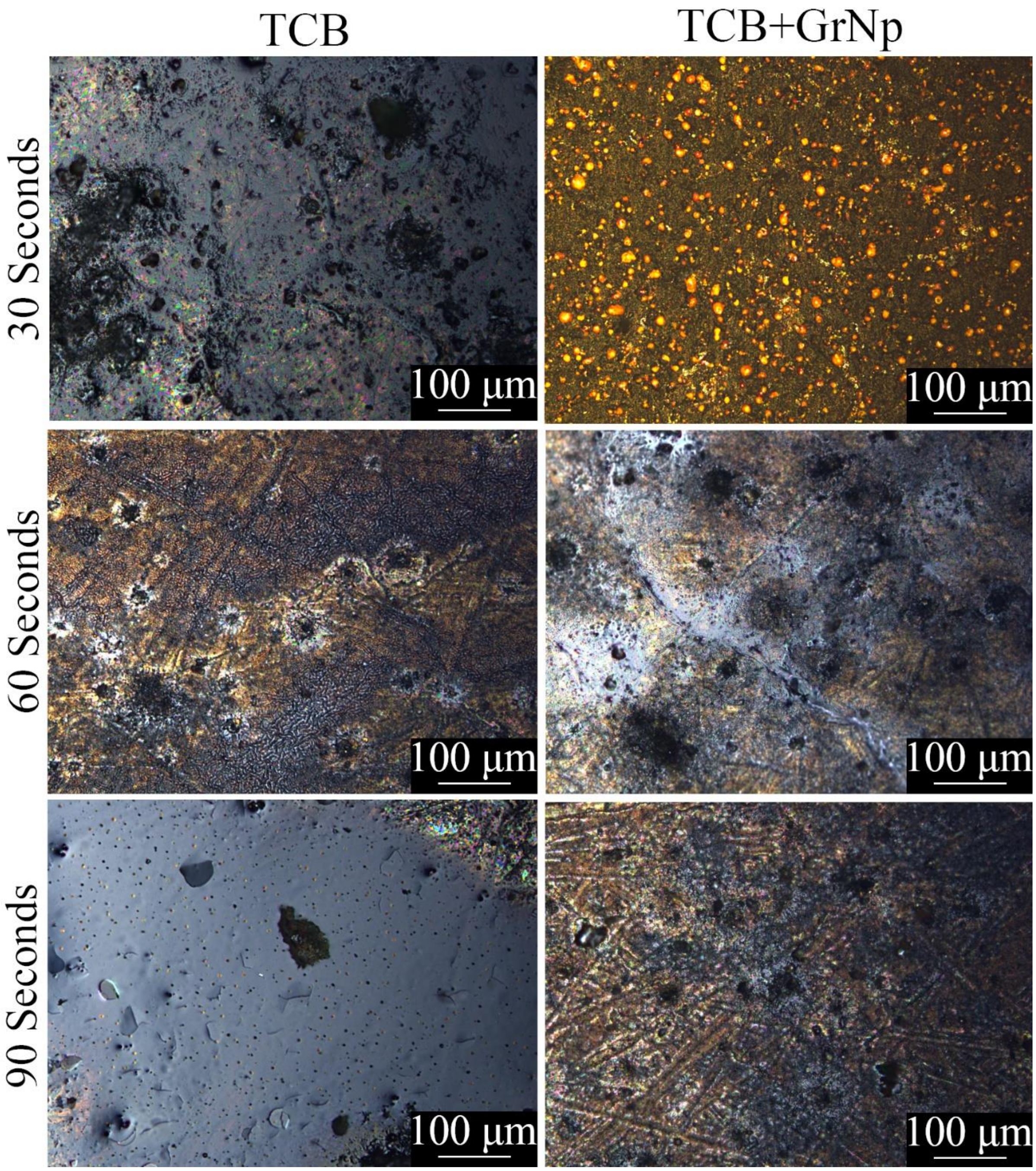

Figure A.10 Optical microscopy of plasma reacted TCB and TCB+GrNp on mild steel substrate, at 130 W for varying durations (specified along the vertical axis of the figure).

SEM of Films Produced from Other Plasma Parameters

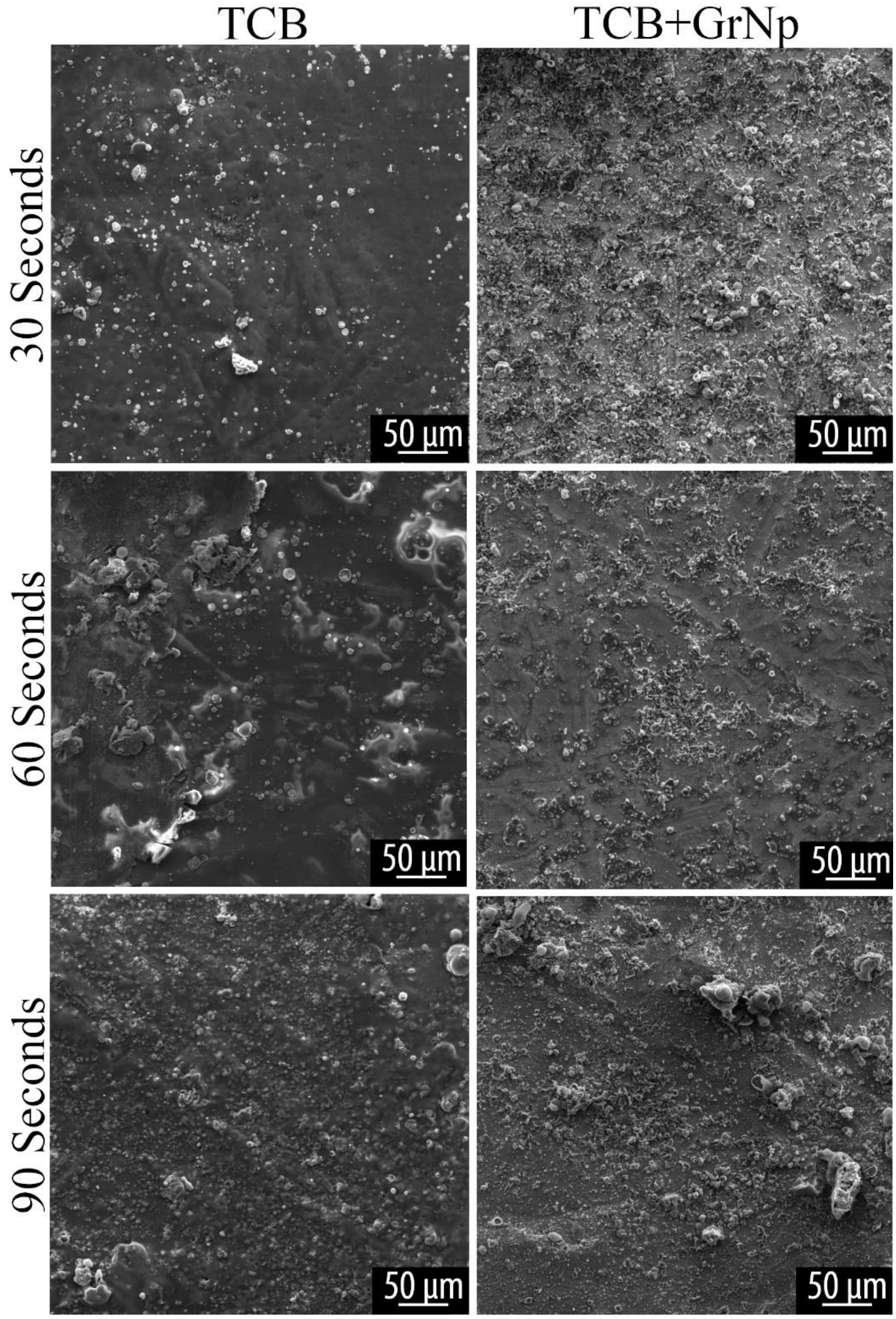

Figure A.11 SEM of plasma reacted TCB and TCB+GrNp on mild steel substrate, at 90 W for varying durations (specified along the vertical axis of the figure).

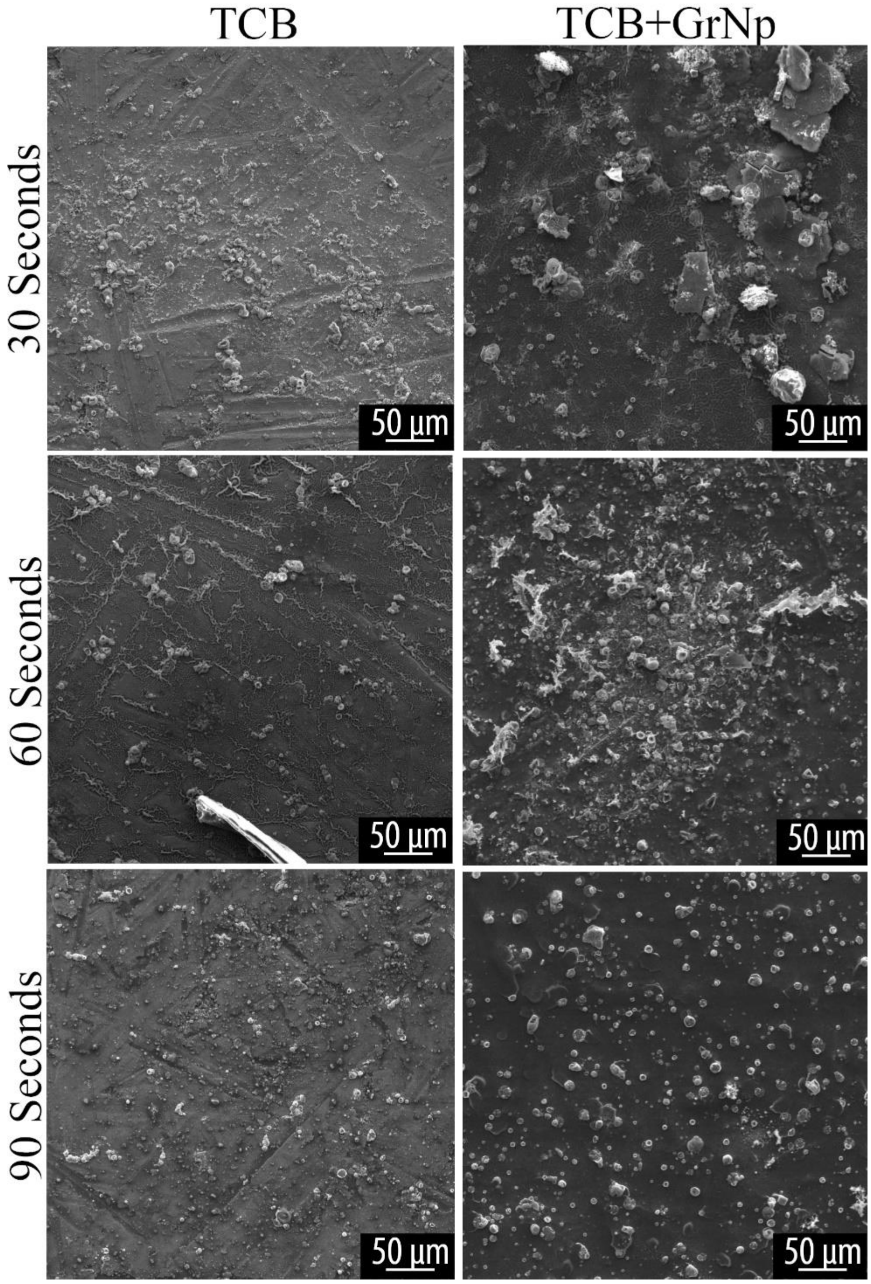

Figure A.12 SEM of plasma reacted TCB and TCB+GrNp on mild steel substrate, at 130 W for varying durations (specified along the vertical axis of the figure).

Water Contact Angle of Films Produced from Other Plasma Parameters

Table A.4 Contact angles for all samples.

90 W	TCB (°)	TCB+GrNp (°)
30 s	74 ± 3	126 ± 11
60 s	70 ± 8	124 ± 6
90 s	77 ± 7	85 ± 3

110 W	TCB (°)	TCB+GrNp (°)
30 s	75 ± 5	114 ± 9
60 s	85 ± 5	151 ± 3
90 s	88 ± 3	80 ± 8

130 W	TCB (°)	TCB+GrNp (°)
30 s	85 ± 5	143 ± 4
60 s	95 ± 7	82 ± 7
90 s	90 ± 3	84 ± 15

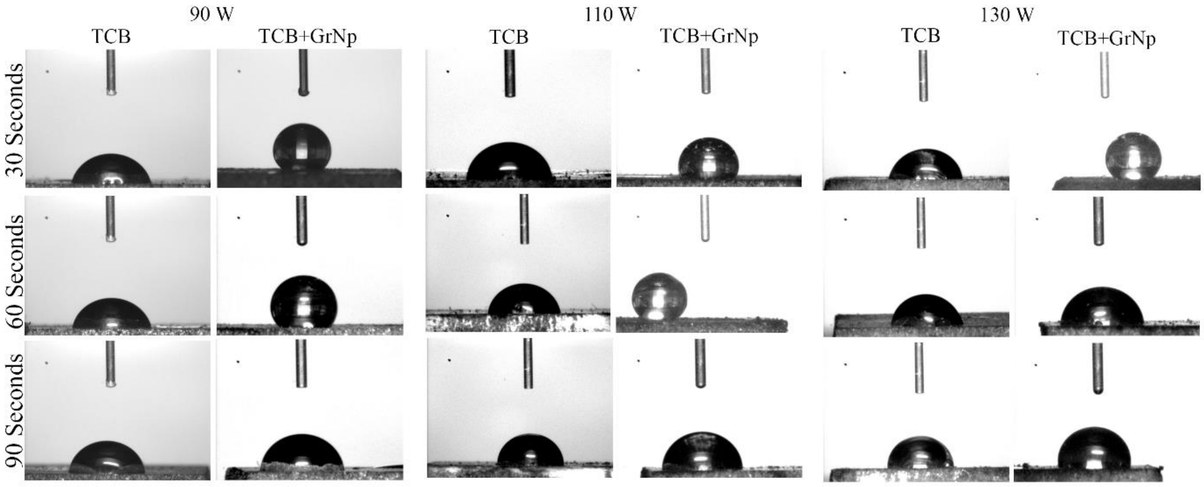

Figure A.13 Water contact angle images for all samples produced.

FTIR of TCB+GrNp Film

Fourier-transform infrared spectroscopy (FTIR, Bruker ATR) was used to chemically characterize organic films over the range of 400 cm^{-1} to 4000 cm^{-1} wavenumbers [2].

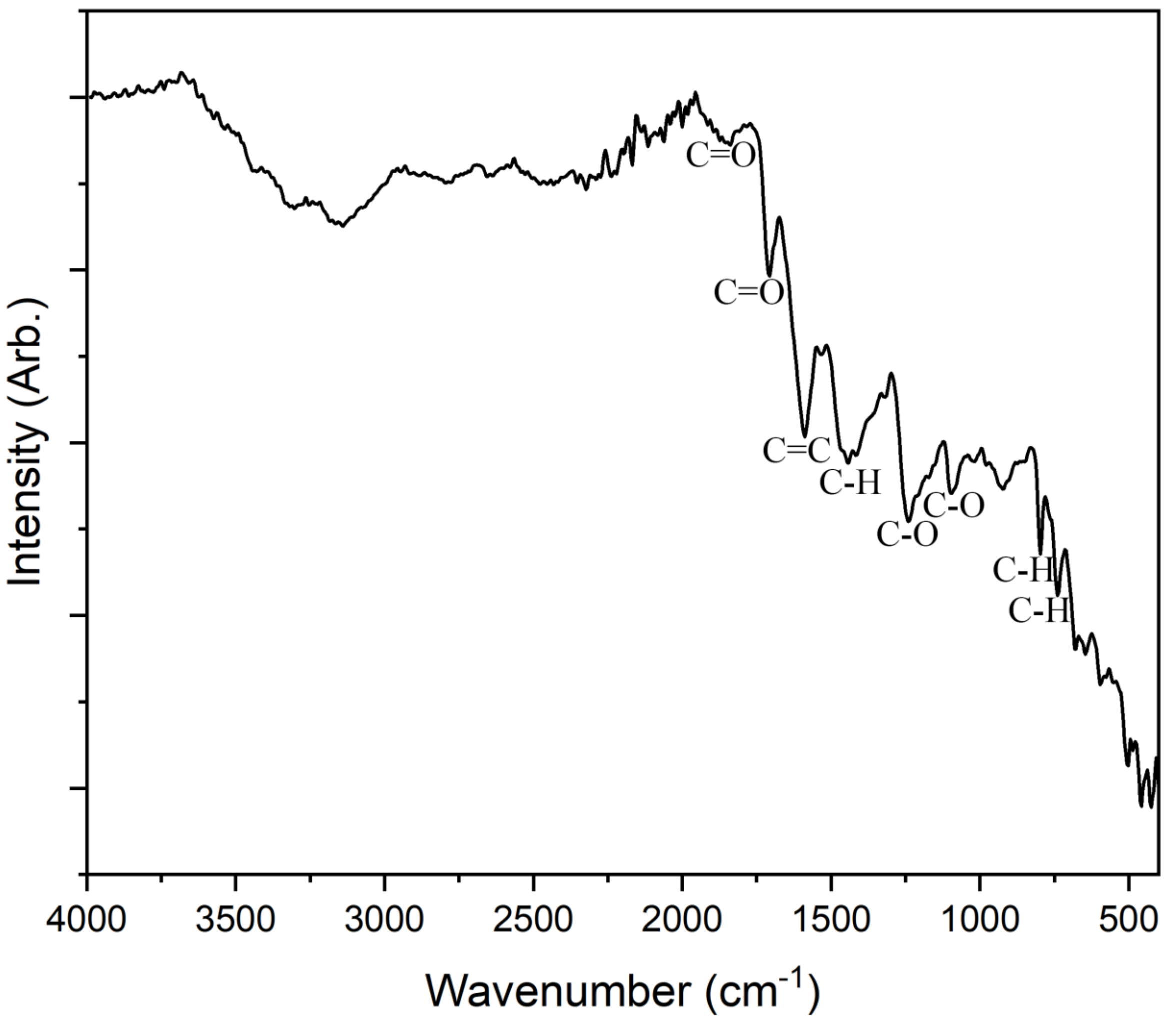

Figure A.14 FTIR of the TCB+GrNp film produced at 110 W 60 s plasma.

Literature of Superhydrophobic Films

Table A.5 Literature of superhydrophobic coatings produced, detailing the water contact angle (WCA) of films tested with corrosion tests including potentiodynamic polarization (PDP), and electrochemical impedance spectroscopy (EIS).

Coating	Substrate	WCA (°)	Corrosion Protective	Corrosion Test Details	Reference
Kerosene	Stainless steel	157 ± 1	No	PDP	[3]
Polythiophene	Stainless steel	152 ± 1	Yes	PDP	[4]
PVC	Glass	151.5	Yes	Acid/Alkali WCA	[5]
Cerium Oxide	Magnesium	153 ± 2	Yes	EIS	[6]
Triazine dithiol	Magnesium	158	Yes	PDP, EIS	[7]
WEP/graphite	Mild steel	151	Yes	Seawater Immersion	[8]
Silane/Fluoropolymer	Stainless steel	166	Yes	PDP	[9]
Silica	Mild steel	155	Yes	EIS, iron dissolution	[10]
Nickel	Stainless steel	150	Yes	PDP	[11]
Polysiloxane/ZnO	Mild steel	166	Yes	PDP, EIS	[12]

Water Contact Angle of Films after Exposure to Acid/Alkaline Solutions

Acid and alkaline solutions were made from dilution of H_2SO_4 and NaOH in distilled water, respectively. The pH3 and pH11 solutions did not appear to have any effect on the water contact angle of the coating over the 1 hour or 12 hour periods. The solution with a pH14 did not have any effect for the first hour, but by 12 hours the film was no longer superhydrophobic, and there was visible delamination of some of the coating. The solution with pH1 reduced the film to no longer hydrophobic after just 1 hour of exposure, with 12 hours causing total delamination of the coating over the whole substrate.

Table A.6 Water contact angles of a TCB+GrNp sample created at 110 W 60 s after exposure to acid/alkaline solutions.

	pH 1 (°)	pH 3 (°)	pH 11 (°)	pH 14 (°)
1 hour	133 ± 1	160 ± 6	164 ± 9	156 ± 3
12 hours	-	158 ± 4	160 ± 4	146 ± 2

Further Water Contact Angle

Further water contact angle measurements were taken of the superhydrophobic (110 W, 60 s) sample. The advancing and receding angles of the sample were measured by a Biolin ThetaFlex. From this the contact angle hysteresis was calculated. Advancing and receding angles measure less than static measurements due to the homogeneity of the films, and the droplet seeking a less hydrophobic region during deposition.

Table A.7 Water contact angles of a TCB+GrNp sample created at 110 W 60 s.

Advancing (°)	Receding (°)	Hysteresis (°)
138.1	133.8	4.3

Cost Analysis of Scale-up

Assuming that the coating could be scaled-up linearly, the process detailed here could produce a 1 m^2 coating using only 18.34 kWh, at a power cost of $2.39 (assuming an electricity price of USD $0.13 per kWh). Scaled-up, the same 1 m^2 coating would require 80 mL of TCB, at an estimated cost of $5.59 USD (99%, Sigma Aldrich). The weight of graphene nanoplates required at 1 wt% would be 0.001168 kg and cost $18.24 USD for a 1 m^2 coating. Scaling the gas flow rate of 200 mL/min for a 10×10 mm^2 coating linearly, we would expect to use 2000 L of gas for a 1 m^2 coating, at a cost of $7.91 USD.

References

1. Fairley, N. CasaXPS VAMAS processing software. *Available from World Wide Web: http://www.casaxps.com* (2018).
2. Aldrich, S. *IR Spectrum Table & Chart*, <https://www.sigmaaldrich.com/AU/en/technical-documents/technical-article/analytical-chemistry/photometry-and-reflectometry/ir-spectrum-table> (2022).
3. Gateman, S. M. *et al.* Corrosion of one-step superhydrophobic stainless-steel thermal spray coatings. *ACS Appl. Mater. Interfaces* **12**, 1523-1532 (2019).
4. de Leon, A. C. C., Pernites, R. B. & Advincula, R. C. Superhydrophobic colloidally textured polythiophene film as superior anticorrosion coating. *ACS Appl. Mater. Interfaces* **4**, 3169-3176 (2012).
5. Kang, Y. *et al.* Preparation of porous super-hydrophobic and super-oleophilic polyvinyl chloride surface with corrosion resistance property. *Appl. Surf. Sci.* **258**, 1008-1013 (2011).
6. Ishizaki, T., Masuda, Y. & Sakamoto, M. Corrosion resistance and durability of superhydrophobic surface formed on magnesium alloy coated with nanostructured cerium oxide film and fluoroalkylsilane molecules in corrosive NaCl aqueous solution. *Langmuir* **27**, 4780-4788 (2011).
7. Kang, Z., Lai, X., Sang, J. & Li, Y. Fabrication of hydrophobic/super-hydrophobic nanofilms on magnesium alloys by polymer plating. *Thin Solid Films* **520**, 800-806 (2011).
8. Gore, P. M., Balakrishnan, S. & Kandasubramanian, B. in *Superhydrophobic Polymer Coatings* 223-243 (Elsevier, 2019).
9. Motlagh, N. V., Birjandi, F. C., Sargolzaei, J. & Shahtahmassebi, N. Durable, superhydrophobic, superoleophobic and corrosion resistant coating on the stainless steel surface using a scalable method. *Appl. Surf. Sci.* **283**, 636-647 (2013).
10. Wu, L.-K., Zhang, X.-F. & Hu, J.-M. Corrosion protection of mild steel by one-step electrodeposition of superhydrophobic silica film. *Corros. Sci.* **85**, 482-487 (2014).
11. Chen, L. J., Chen, M., Di Zhou, H. & Chen, J. M. Preparation of super-hydrophobic surface on stainless steel. *Appl. Surf. Sci.* **255**, 3459-3462 (2008).
12. Qing, Y., Yang, C., Hu, C., Zheng, Y. & Liu, C. A facile method to prepare superhydrophobic fluorinated polysiloxane/ZnO nanocomposite coatings with corrosion resistance. *Appl. Surf. Sci.* **326**, 48-54 (2015).
